# Vessel Wall Inflammation of Takayasu Arteritis Detected by Contrast-Enhanced Magnetic Resonance Imaging: Association with Disease Distribution and Activity

**DOI:** 10.1371/journal.pone.0145855

**Published:** 2015-12-31

**Authors:** Yoko Kato, Masahiro Terashima, Hirokazu Ohigashi, Daisuke Tezuka, Takashi Ashikaga, Kenzo Hirao, Mitsuaki Isobe

**Affiliations:** 1 Department of Cardiovascular Medicine, Tokyo Medical and Dental University, Tokyo, Japan; 2 Cardiovascular Imaging Clinic, Tokyo, Japan; Tokai University, JAPAN

## Abstract

**Aims:**

The assessment of the distribution and activity of vessel wall inflammation is clinically important in patients with Takayasu arteritis. Magnetic resonance imaging (MRI) is a useful tool, but the clinical utility of late gadolinium enhancement (LGE) in Takayasu arteritis has yet to be determined. The aim of the present study was to evaluate the utility of LGE in assessing vessel wall inflammation and disease activity in Takayasu arteritis.

**Methods and Results:**

We enrolled 49 patients with Takayasu arteritis who had undergone 1.5 T MRI. Patients were divided into Active (n = 19) and Inactive disease (n = 30) groups. The distribution of vessel wall inflammation using angiography and LGE was assessed by qualitative analysis. In 79% and 63% of patients in Active and Inactive groups, respectively, greater distribution of vessel wall inflammation was observed with LGE than with conventional angiography. MRI values of pre- and post-contrast signal-to-noise ratios (SNR), SNR increment (post-SNR minus pre-SNR), pre- and post-contrast contrast-to-noise ratios (CNR), and CNR increment (post-CNR minus pre-CNR) were evaluated at arterial wall sites with the highest signal intensity using quantitative analysis of post-contrast LGE images. No statistically significant differences in MRI parameters were observed between Active and Inactive groups. Contrast-enhanced MRI was unable to accurately detect active disease.

**Conclusion:**

Contrast-enhanced MRI has utility in detecting the distribution of vessel wall inflammation but has less utility in assessing disease activity in Takayasu arteritis.

## Introduction

Takayasu arteritis causes vessel wall inflammation of the aorta and its major branches [1]. The pathogenesis of Takayasu arteritis remains unclear, with inflammation typically remitting and relapsing [2,3]. As early detection of relapses can improve the control of inflammation, the assessment of disease activity is clinically important. The National Institutes of Health (NIH) criteria proposed by Kerr *et al*. [4] are currently used for the assessment of disease activity on the basis of clinical, laboratory, and angiographic evidences. However, current methods for the clinical assessment of disease severity remain technically challenging with inadequate efficacy.

Recent reports have described the utility of imaging tests such as positron emission tomography-computed tomography (PET-CT), [5] computed tomography angiography (CTA), [6,7] and echocardiography [8–12] in the assessment of disease activity in patients with Takayasu arteritis. Magnetic resonance imaging (MRI) is increasingly being utilized for this purpose. Previous studies in small numbers of patients have demonstrated late gadolinium enhancement (LGE) of MRI is able to detect edema of the arterial wall caused by inflammation [2, 13–16].

Quantitative LGE analysis allows the detailed assessment of the coronary vessels [17–19], in addition to the larger arteries. A previous study of quantitative vessel wall analysis in 23 Takayasu arteritis patients used gadofosveset, an intravascular contrast agent. The kinetics of gadofosveset are quite different from widely-used gadolinium agents [15,19]. Further, intravascular gadolinium contrast agent is not currently available in Japan.

The aim of the present study was to evaluate the utility of LGE with a widely used gadolinium-based contrast agent in detecting vessel wall inflammation and its activity in a relatively large number of patients with Takayasu arteritis.

## Materials and Methods

### Patient population

We retrospectively reviewed 57 consecutive patients with Takayasu arteritis who underwent aortic MRI between March 2011 and May 2012 at our institution. The hospital records of the patients’ data were de-identified and analyzed anonymously. Patients were divided into two groups (Active and Inactive) according to disease activity based on the following criteria: 1) Active patients required increased dose or change of immunosuppressant medication class within the previous 2 years; and 2) Inactive patients had not received medications or received anti-platelet, immunosuppressive therapy, or both without dose modification within the previous 2 years. These criteria ensured that only patients with inactive disease were included in the Inactive group, although inactive patients may have been included in the Active group. To address potential misgrouping, we compared patients from the Active group still within the first episode of inflammation, i.e., those who had yet to achieve remission (First-episode group), with patients in the Inactive group. In addition, we assessed the disease activity using Indian Takayasu’s Arteritis Activity Score (ITAS2010) [20] to add information of the disease activity. Furthermore, we conducted a sub-study on patients regrouped according to the traditional NIH criteria [4].

**Fig 1:**
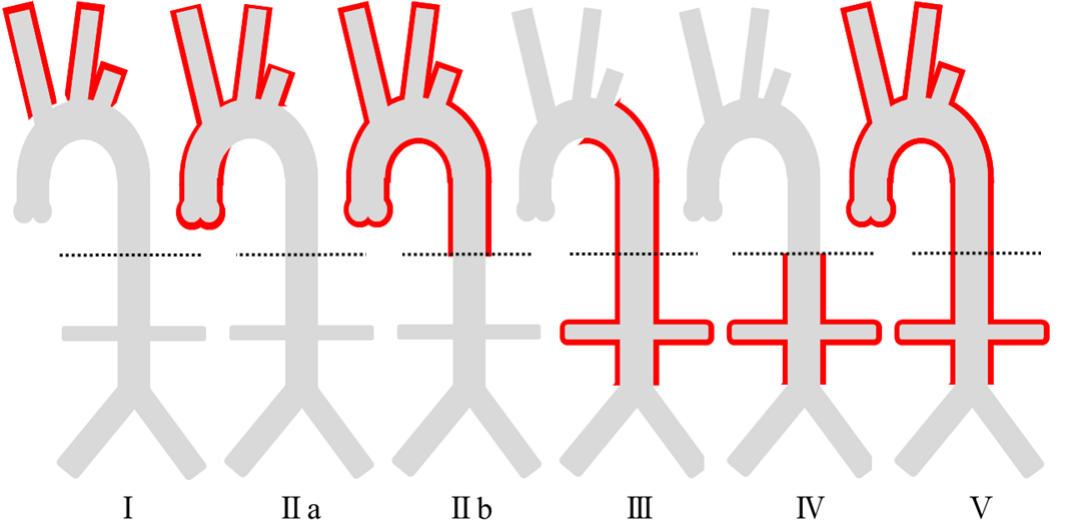
Angiographic classification of Takayasu arteritis. The dotted lines refer to the diaphragms.

The distribution of vessel wall inflammation was categorized as Type I to Type V according to the angiographic classification of Hata *et al*.[1,21] (Fig 1) on the basis of the location of inflammatory changes affecting the arterial lumen. This classification, designated “classical,” was defined as follows: Type I primarily involved branches of the aortic arch; Type IIa involved the ascending aorta, aortic arch, and associated branches; Type IIb involved the ascending aorta, aortic arch with associated branches, and the thoracic descending aorta; Type III involved the thoracic descending aorta, abdominal aorta, and/or renal arteries; Type IV affected only the abdominal aorta and/or renal arteries; and Type V included patients with combined features of Types IIb and IV. In patients who had undergone catheter angiography, CTA, or ultrasonography (US), we assessed the classification based on information regarding the arterial lumen gained from these imaging modalities. Patients who underwent MRI imaging only were classified according to morphological images of the arterial lumen.

All participants were provided with written information about the study prior to consent being obtained. Verbal consent was obtained by doctors of the outpatient clinic (Y.K. and M.I.) and documented in a list. Written consent was not obtained because it was a retrospective observational study with images and clinical datum taken prior to this study and no new procedures were performed on the patients. The oral informed consent process was approved by the Institutional Ethics Review Committee of the Tokyo Medical and Dental University. The study was registered in the University Hospital Medical Information Network Clinical Trials Registry (UMIN-CTR; Clinical Trial registration number, R000017533; trial ID, UMIN000015085; UMIN-CTR URL, http://www.umin.ac.jp/ctr/index-j.htm).

### MRI protocol

Imaging was performed using a 1.5-T MR scanner (Achieva Dual, Philips Healthcare) equipped with a torso cardiac 32-channel coil. The MRI coil was positioned over the neck and chest to acquire images of the carotid artery to the thoracic aorta before repositioning over the abdomen to acquire images of the abdominal aorta. ECG triggering was used throughout the present study.

For morphological investigations, a triggered angiography non-contrast enhanced (TRANCE) long sensitivity encoding (SENSE) sequence was used. Image acquisition parameters were adjusted for individual patients to produce images of optimal quality. The mean values of image acquisition parameters were as follows: echo time (TE), 54.2 ± 1.05 ms; repetition time (TR), 852 ± 124 ms; field of view (FOV), 35.0 ± 0.0 × 35.0 ± 0.0 cm; and voxel size, 1.5 ± 0.0 × 2.4 ± 2.6 × 5.9 ± 0.42 mm.

Next, the LGE sequence using gadopentetate dimeglumine (Magnevist, Bayer) as the MRI contrast medium was performed. The LGE sequence used was 3D/LGE SENSE. Image acquisition parameters were adjusted to provide the shortest TE and TR for each patient. First, images were acquired prior to contrast infusion. After intravenous infusion of a 0.15 mmol/kg bolus of contrast medium, a Look-Locker inversion-recovery sequence in the coronal view was performed to assess the blood pool null point. LGE images were then acquired using the optimal inversion time. The Look-Locker sequence followed by LGE image acquisition was serially repeated for approximately 30 minutes according to previous studies of LGE analyses of the coronary arteries and in patients with Takayasu arteritis [13–18]. LGE images with the best enhancement were selected for qualitative and quantitative analyses. The mean values of image acquisition parameters for pre-contrast images were as follows: TE, 54.2 ± 1.06 ms; TR, 855 ± 123 ms; FOV, 35.0 ± 0.0 × 35.0 ± 0.0 cm; and voxel size, 1.5 ± 0.0 × 2.37 ± 2.57 × 5.90 ± 0.42 mm. The mean values of image acquisition parameters for LGE were as follows: TE, 3.03 ± 0.15 ms; TR, 6.16 ± 0.29 ms; FOV, 48.8 ± 3.32 × 48.8 ± 3.32 cm; and voxel size, 1.3 ± 0.10 × 1.7 ± 0.24 × 10.0 ± 0.28 mm. For analyses of MRI images, a 3D workstation (Ziostation2 and zioTerm2009, Ziosoft, Tokyo, Japan) was used.

### Qualitative LGE distribution analysis

LGE distribution patterns were classified as Types I–V in the same manner as the angiographic classification proposed by Hata *et al*. described above [1,21,22]. We evaluated the degree of correspondence between the LGE-based and classical angiographic classifications of Active and Inactive group patients. Two observers (Y.K. and M.T.) evaluated the LGE distribution pattern. Disagreements between the two observers were resolved by means of consensus reading.

### Quantitative image analysis

**Fig 2:**
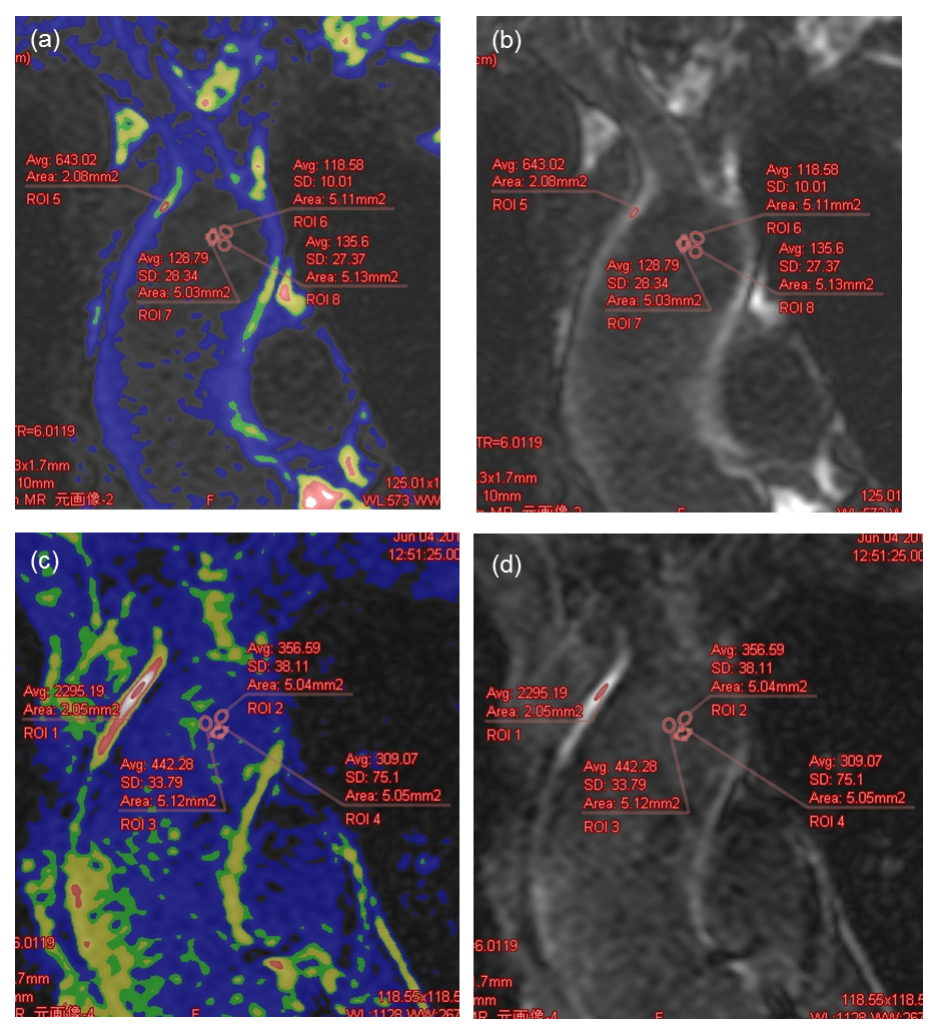
MRI imaging. (a) Representative pre-contrast image with color map. (b) Representative pre-contrast image. (c) Representative post-contrast image with color map. The site of the aortic wall site with the highest SI was determined using this image. (d) Representative post-contrast image. SI, signal intensity; LGE, late gadolinium enhancement.

The signal intensity (SI) of the arterial wall (SI_vessel_) was assessed by positioning region of interest (ROI) on the region of the aortic wall with the highest SI. Color mapping was used to determine the site with the highest SI in post-contrast image, and the corresponding site was used for pre-contrast image. Average SI within ROI was determined. To quantify SI derived from noise signals, three ROIs were drawn in the center of the aorta adjacent to the aortic wall site of SI_vessel_. The mean signal intensity of the three aortic ROIs (SI_noise_) and the mean standard deviation (SD) (SD_noise_) in pre- and post-contrast infusions were measured (Fig 2).

The signal-to-noise ratio (SNR = SI_vessel_/SD_noise_) and contrast-to-noise ratio [CNR = (SI_vessel_ − SI_aorta_)/SD_noise_] were calculated from pre-contrast and post-contrast LGE images, referred to as pre-SNR and post-SNR, and pre-CNR and post-CNR, values accordingly. To assess changes in SNR and CNR values from pre-contrast images, the SNR increment (post-SNR minus pre-SNR) and CNR increment (post-CNR minus pre-CNR) were calculated. MRI parameters were compared between the Active and Inactive groups.

To determine inter-observer and intra-observer reproducibility, two experienced observers (Y.K. and M.T.) independently performed quantitative image analyses. For inter-observer analyses, ten patients were randomly selected and the results provided by observers were compared. For intra-observer analysis, measurements were performed twice by the same observer (Y.K.). Inter- and intra-observer variability were evaluated using Bland-Altman analysis [23].

### Statistical analysis

All statistical analyses were performed using SPSS software (SPSS for Windows version 22.0, Chicago, IL, USA). P-values < 0.05 were considered statistically significant. Continuous variables are expressed as the mean ± standard deviation (SD). Patient characteristics were compared between the Active and Inactive groups using the chi-square test for categorical values and the Student’s *t-*test for continuous values. Statistical comparisons of MRI parameters between the Active and Inactive groups were performed using the Mann–Whitney test. Bland-Altman analysis was used to assess inter- and intra-observer variability.

## Results

### Patient characteristics

We retrospectively evaluated 57 patients with Takayasu arteritis (two males, 55 females). Of these 57, eight patients were excluded due to insufficient data such as lack of complete MRI data set. Thus, 49 patients were included in the final data analysis. Patient characteristics are shown in Table 1. According to disease activity, 19 and 30 patients were assigned to the Active and Inactive disease groups, respectively. Of the 19 patients in the Active group, six were also included in the first-episode group. Statistically significant differences in age (31.7 ± 11.0 vs. 47.9 ± 14.0 years; P < 0.01), duration of disease (82.7 ± 97.0 vs. 205 ± 151 months; P < 0.01), serum CRP level (0.88 ± 1.3 vs. 0.13 ± 0.16 mg/dL; P = 0.02), and ITAS2010 score (5.00 ± 2.75 vs. 2.47 ± 3.27; P < 0.01) were observed between patients in the Active and Inactive groups. Furthermore, statistically significant differences in age, duration of disease, and ITAS2010 score were observed between patients in the First-episode and Inactive groups.

**Table 1:** Patient characteristics.

Characteristics	Active group (n = 19)	Inactive group (n = 30)	First-episode group (n = 6)	P-value (Active vs. Inactive)	P-value (First-episode vs. Inactive)
Female, n (%) ^a^	18 (94.7)	29 (96.7)	6 (100)	0.75	0.66
Mean age (years) ^a^	31.7 ± 11.0	47.9 ± 14.0	32.8 ± 8.80	<0.01**	0.016*
Duration of Disease (months) ^a^	82.7 ± 97.0	205 ± 151	11.5 ± 7.35	<0.01**	<0.01**
Serum CRP level (mg/dl) ^a^	0.88 ± 1.3	0.13 ± 0.16	0.54 ± 0.49	0.022*	<0.10
ITAS2010 ^a^	5.00 ± 2.75	2.47 ± 3.27	5.83 ± 2.71	<0.01**	0.024*
Time to LGE image acquisition after contrast infusion (min) ^a^	24.6 ± 6.88	28.5 ± 6.96	23.5 ± 5.43	0.062	0.11

CRP, C-reactive protein; LGE, late gadolinium enhancement

^a^ Values represent the mean ± S.D. except for females, which is present as a count and percentage.

* P <0.05

** P <0.01.

**Fig 3:**
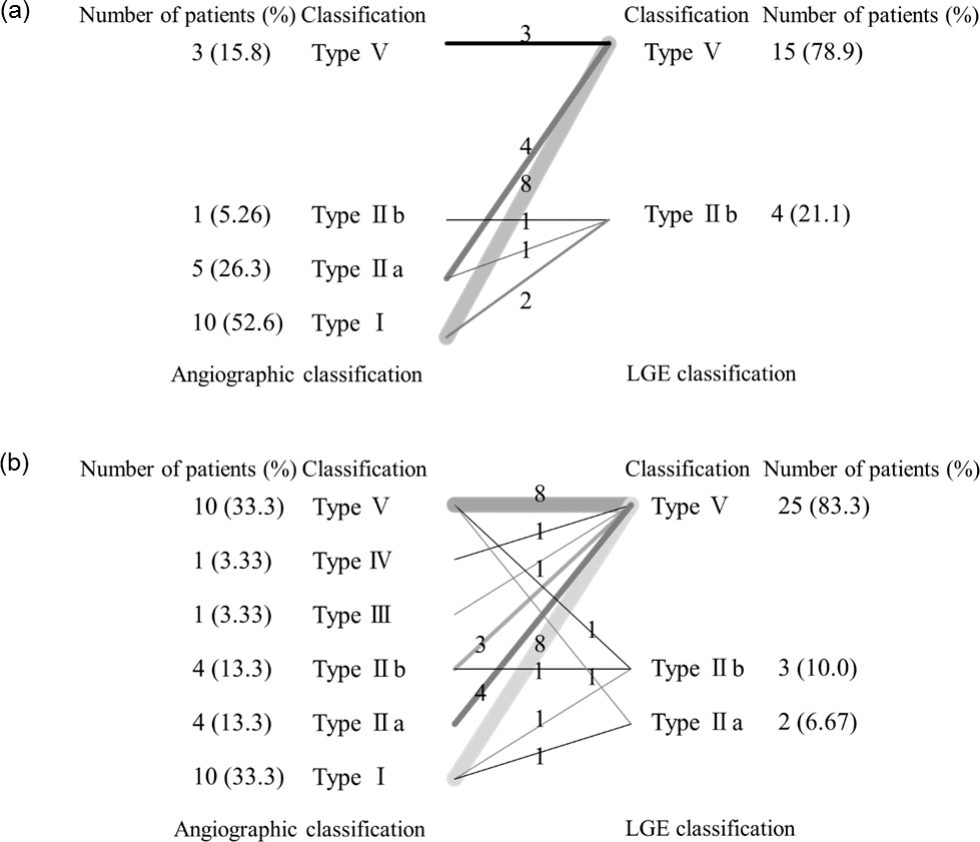
Comparison of the distribution of Takayasu arteritis according to angiography and LGE. (a) Comparisons in patients with active disease. (b) Comparisons in patients with inactive disease. The numbers and percentages of patients included in each classification type are presented in the left and right columns (arabic numerals). Lines connect the angiographic classification (roman numerals in the left column) with the LGE distribution classification (roman numerals in the right column). The number on the line and thickness of the line reflect the number of patients with each type of disease classification. For example, eight patients in panel (a) were classified as having Type I disease according to the angiographic classification and Type V disease according to the LGE classification. The LGE distribution was identical or larger than the angiographic disease distribution in 100% and 93% of patients with active and inactive disease, respectively. LGE, late gadolinium enhancement.

Classical angiographic classifications according to imaging modalities other than MRI were available in 15 (79%) and 28 (93%) of patients in the Active and Inactive groups, respectively. The distribution of disease classifications was consistent with that reported by Moriwaki *et al*., [22] namely types I, IIa, and V were dominant among Japanese patients (Fig 3A and 3B).

### Qualitative LGE distribution analysis

All patients presented LGE positive in the aorta. LGE distribution and classical angiographic classifications were in agreement in four (21%) patients in the Active group and nine (30%) patients in the Inactive group. The LGE distribution was larger than the classical angiographic classification in 15 (79%) and 19 (63%) patients in the active and inactive groups, respectively. Therefore, LGE covered an identical or larger area compared with that in the classical angiographic classification in 100% and 93% patients in the Active and Inactive groups, respectively (Fig 3A and 3B).

### Quantitative Image analysis

**Fig 4:**
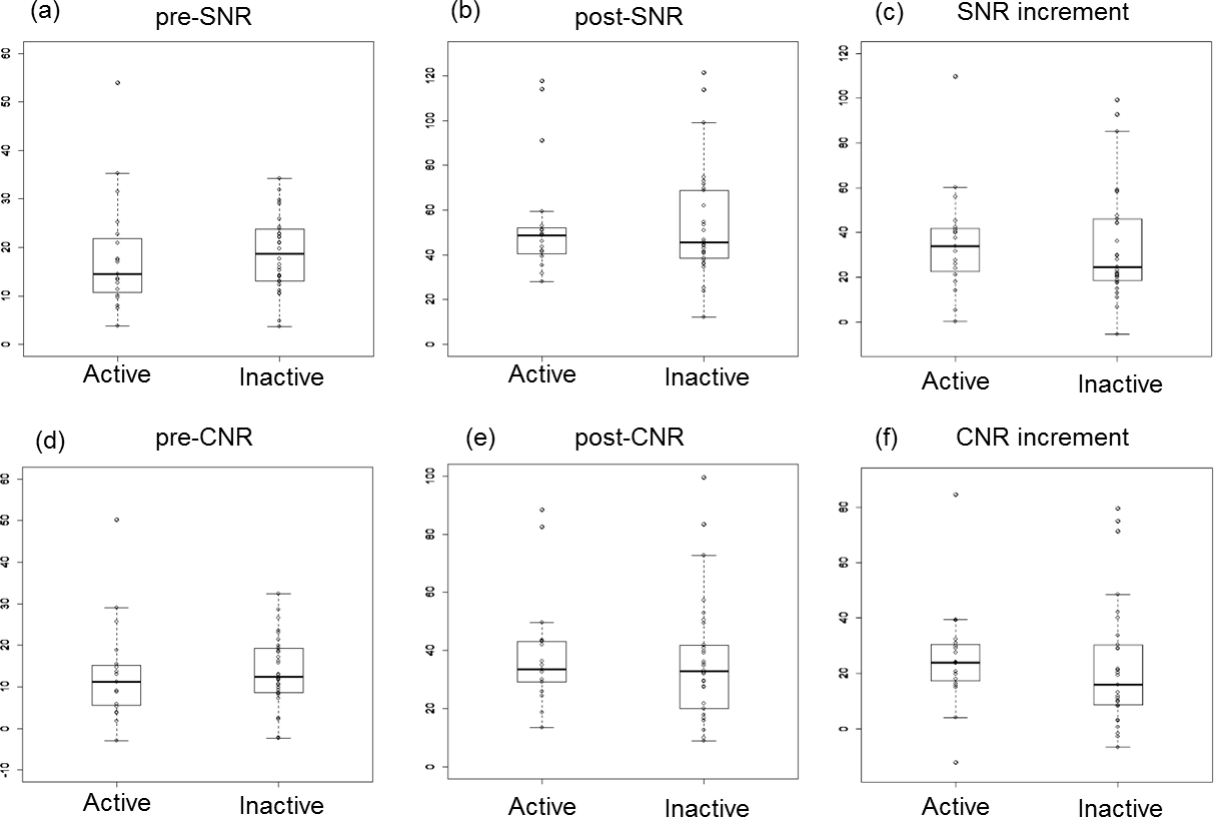
Comparisons of MRI parameters between patients with Active and Inactive patients. Dots on box plots represent the distribution of cases. (a) (b) (c) Comparisons of pre-SNR, post-SNR, and SNR increment. (d) (e) (f) Comparisons of pre-CNR, post-CNR, and CNR increment. No statistically significant difference in any MRI parameter was observed between the groups. SNR: signal-to-noise ratio, CNR: contrast-to-noise ratio.

Fig 4 shows boxplot comparisons of the MRI parameters of patients in the Active and Inactive groups. Dots shown on the box plots represent the distribution of cases. No statistically significant differences in any MRI parameter were observed between the Active and Inactive groups including pre-SNR (14.6 vs. 18.7; U = 238; P = 0.34), pre-CNR (11.2 vs. 12.4; U = 250; P = 0.47), post-SNR (48.7 vs. 45.5; U = 284; P = 0.98), post-CNR (33.6 vs. 32.8; U = 252; P = 0.50), SNR increment (34.0 vs 24.6; U = 259; P = 0.59), or CNR increment (23.9 vs. 15.9; U = 221; P = 0.19).

No statistically significant differences in any MRI parameter were observed between patients in the First episode group and patients in the Inactive group including pre-SNR (10.7 vs. 18.7; U = 53; P = 0.12), pre-CNR (7.27 vs. 12.4; U = 62; P = 0.24), post-SNR (39.5 vs. 45.5; U = 58; P = 0.17), post-CNR (28.0 vs. 32.8; U = 74; P = 0.50), SNR increment (25.0 vs. 24.6; U = 78; P = 0.61), or CNR increment (20.3 vs. 15.9; U = 84; P = 0.80).

**Fig 5:**
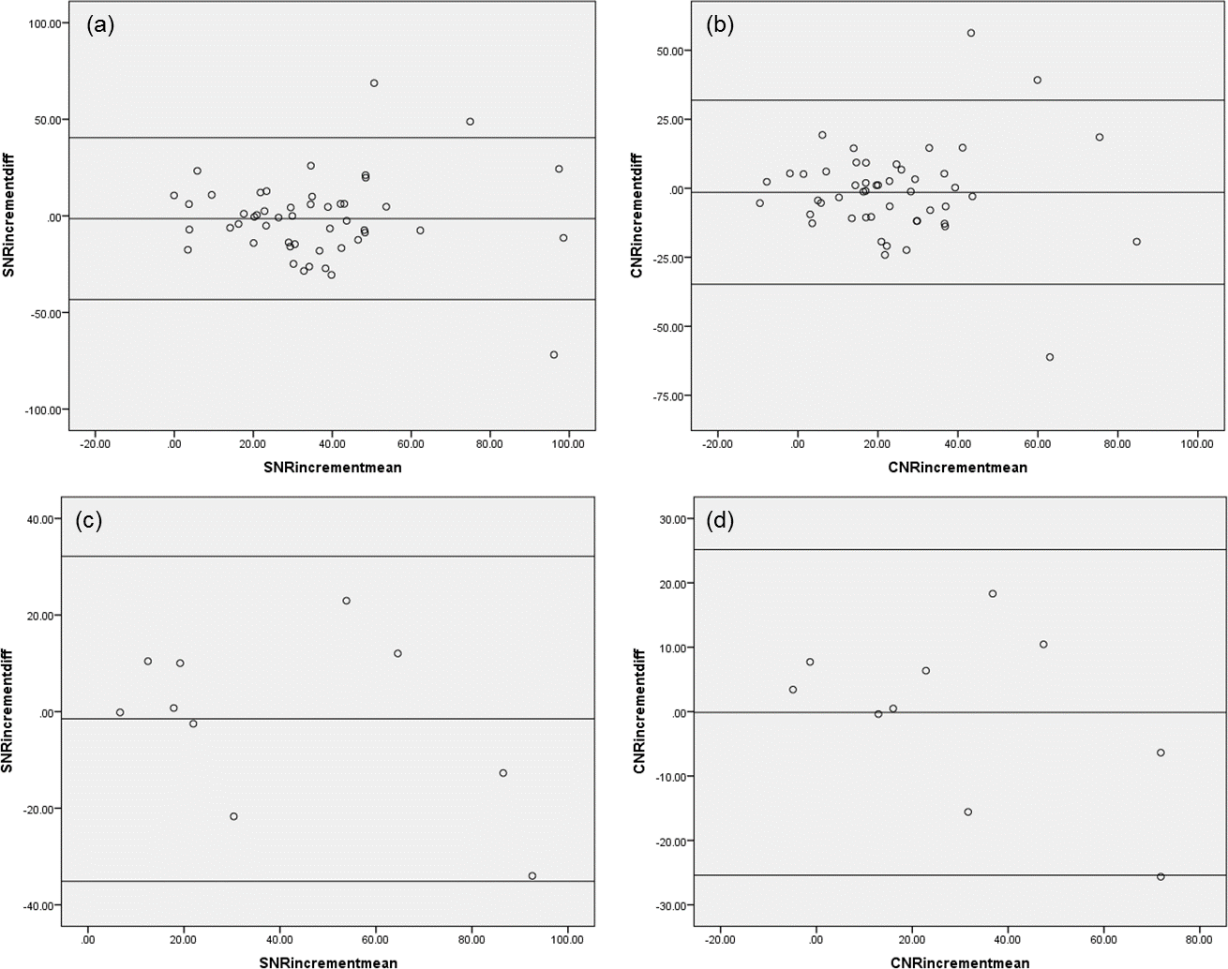
Bland-Altman plot of intra-observer and inter-observer analyses. (a) Intra-observer variability of SNR increment analysis. (b) Intra-observer variability of CNR increment analysis. (c) Inter-observer variability of SNR increment analysis. (d) Inter-observer variability of CNR increment analysis. Good reproducibility of both intra- and inter-observer analyses was observed. SNR, signal-to-noise ratio; CNR, contrast-to-noise ratio.

Intraobserver and interobserver analyses demonstrated good reproducibility of the quantitative analysis. Fig 5A and 5B shows Bland-Altman Plots illustrating the intra-observer variability observed for SNR increment and CNR increment assessments. For SNR increment, the 95% limits of agreement ranged from −43.3 to 40.4, and the mean value of the differences was −1.42. For CNR increment, the 95% limits of agreement ranged from −34.8 to 31.9, and the mean value of the differences was −1.44. The inter-observer variability of SNR increment and CNR increment assessment were as follows: for SNR increment, the 95% limits of agreement ranged from −35.2 to 32.2, with mean value of the differences found to be −1.48; and for CNR increment, the 95% limits of agreement ranged from −25.4 to 25.2, with the mean value of the difference found to be −0.12 (Fig 5C and 5D).

### Sub-study

Patients were regrouped according to the traditional NIH criteria. Six patients in the Active group according to our definition were moved to the Inactive group, because the active inflammation was not occurring at the time of MRI, even though the episode of active inflammation and modification of treatment occurred within 2 years prior to the MRI. Consequently, 13 patients were included in the Active group and 39 patients were included in the Inactive group. The reassessed ITAS2010 score was significantly higher in the Active group, which was the same result as when our criteria were used (5.38 ± 2.96 vs. 2.75 ± 3.16, P = 0.012*). Although we regrouped the patients, no statistically significant differences in any of the MRI parameters were observed between patients in the Active and Inactive groups, including pre-SNR (13.6 vs. 18.7; U = 169; P = 0.14), pre-CNR (9.00 vs. 12.9; U = 171; P = 0.15), post-SNR (46.2 vs. 46.2; U = 217; P = 0.70), post-CNR (32.8 vs. 33.4; U = 217; P = 0.70), SNR increment (31.6 vs. 26.3; U = 217; P = 0.70), and CNR increment (24.2 vs. 17.6; U = 171; P = 0.15).

## Discussion

We evaluated the distribution of vessel wall inflammation and disease activity in 49 patients with Takayasu arteritis by contrast-enhanced MRI. LGE was found to provide greater sensitivity in detecting vessel wall inflammation than traditional angiography. However, LGE had no utility in assessing disease activity as statistically significant differences in MRI-based parameters were observed between patients in the Active and Inactive groups.

LGE detected vessel wall inflammation over a larger distribution than traditional angiography in more than half of patients in both the Active and Inactive groups. This is attributed to the limitation of angiographic classification based only on the diameter of arterial lumen. MRI can visualize the vessel wall directly and has the potential to detect pathologic changes of the arterial wall independent of luminal diameter. The detection of subclinical disease in affected vessels by MRI is beneficial for disease management and contributes to careful monitoring especially in patients that have been recently diagnosed.

It was not possible to determine disease activity using LGE quantification data. The lack of specificity of the conventional gadolinium-based contrast agent may have contributed to this result. The gadolinium contrast agent used in the present study rapidly distributes into the extracellular space and is quickly washed out of normal tissues. LGE may be associated with an increased distribution volume (as observed with fibrosis and neovascularization) in vessel walls or with increased vascular permeability (as may occur with inflammation). Increased vascular permeability may be associated with active inflammation in Takayasu arteritis, whereas fibrosis and neovascularization may be associated with an inactive inflammatory status. However, LGE was unable to distinguish two conditions based only on LGE intensity. This may explain the lack of a positive association between disease activity and LGE in the present study. To assess disease activity, it may be better to combine LGE with other imaging modalities such as PET-CT [5], CTA [6,7], or echocardiography [8–12]. We specifically recommend PET-CT due to higher sensitivity and specificity in detecting disease recurrence.

The present study differed from a previous report by Papa *et al*. [15] that used an intravascular contrast agent with different kinetics to the commonly used gadolinium-based contrast agent. Our study used gadopentetate dimeglumine, which is widely used clinically. To date, the present study represents the largest population study to assess LGE in Takayasu arteritis patients using a widely used gadolinium contrast agent. Further studies are necessary to determine the best gadolinium-based contrast agent for Takayasu arteritis. Vessel wall inflammation-targeted MRI contrast agents such as elastin-specific [24], collagen-specific [25], or macrophage-targeted agents [19,26,27] may allow more precise evaluation of disease activity in patients with Takayasu arteritis.

There are some limitations to this study. First, a fat saturation MRI sequence was not used. Therefore, the SI of the aortic wall may have been influenced by the partial volume of perivascular fat despite the comparison of images pre-and post- contrast agent administration and the careful positioning of ROI within the aortic wall. Second, we defined active disease differently to previous reports. Our intent was to provide a conservative estimate of disease inactivity; however, the definition may have contributed to over-representation of patients with inactive disease in the Active group. To reduce this potential misgrouping, we conducted a study of patients currently suffering a first episode of inflammation and demonstrated that there was no statistically significant difference between these patients and patients with inactive disease. Furthermore, we conducted a sub-study, which regrouped the patients according to the traditional NIH criteria, although the results did not change. This sub-study strongly supports our initial result, which suggested that cardiac MRI should not be used to assess inflammation activity in Takayasu arteritis. Finally, we used only LGE data on contrast-enhanced MRI for the present study. Different MRI techniques such as dynamic perfusion or T2 weighted images may contribute to assess the disease activity.

In conclusion, the findings of the present study demonstrated LGE in contrast-enhanced MRI has utility in detecting the extent of vessel wall inflammation in patients with Takayasu arteritis. However, inflammation activity in Takayasu arteritis may be difficult to determine using LGE with widely-used gadolinium-based contrast agents alone. Further study using different MRI techniques is necessary to detect the disease activity in Takayasu arteritis.

## Supporting Information

S1 Table Patient characteristics for 49 patients. NIH, National Institutes of Health; ITAS, Indian Takayasu’s Arteritis Activity Score; CRP, C-reactive protein.
(DOCX)

S2 Table Detailed results of qualitative and quantitative analysis for 49 patients. LGE, late gadolinium enhancement; SNR, signal-to-noise ratios; CNR, contrast-to-noise ratios.
(DOCX)
