# Supplementary material for: Vessel Wall Inflammation of Takayasu Arteritis Detected by Contrast-Enhanced Magnetic Resonance Imaging: Association with Disease Distribution and Activity
Source: PLoS One. 2015 Dec 31;10(12):e0145855. doi: 10.1371/journal.pone.0145855 (PMC4700986; doi:10.1371/journal.pone.0145855)
Supplement: S1 Table — NIH, National Institutes of Health; ITAS, Indian Takayasu’s Arteritis Activity Score; CRP, C-reactive protein. (DOCX) [file pone.0145855.s001.docx]

**S1 Table. Patient characteristics for 49 patients.**

| Patient No. | Sex | Age (years) | Disease activity assessed with our criteria | Disease activity assessed with NIH criteria | ITAS2010 score | Duration of disease (months) | Serum CRP level (mg/dl) |
| --- | --- | --- | --- | --- | --- | --- | --- |
| 1 | F | 20 | Active | Inactive | 3 | 77.1 | 0.22 |
| 2 | F | 52 | Active | Inactive | 5 | 401.5 | 1.17 |
| 3 | F | 34 | Active | Active | 3 | 220.6 | 0.1 |
| 4 | F | 21 | Active | Active | 2 | 121.3 | 0.8 |
| 5 | F | 23 | Active | Active | 11 | 37.3 | 0.5 |
| 6 | F | 49 | Active | Active | 3 | 69.3 | 3.56 |
| 7 | M | 20 | Active | Active | 3 | 39.2 | 4.97 |
| 8 | F | 21 | Active | Active | 8 | 48.6 | 0.46 |
| 9 | F | 28 | Active | Inactive | 6 | 150.9 | 0.14 |
| 10 | F | 26 | Active | Inactive | 7 | 144.2 | 0.07 |
| 11 | F | 52 | Active | Inactive | 1 | 52.3 | 0.28 |
| 12 | F | 31 | Active | Inactive | 3 | 43.0 | 1.11 |
| 13 | F | 28 | Active | Active | 5 | 97.7 | 0.03 |
| 14 | F | 22 | Active (first episode) | Active | 6 | 19.4 | 1.14 |
| 15 | F | 45 | Active (first episode) | Active | 4 | 0.9 | 0.03 |
| 16 | F | 33 | Active (first episode) | Active | 4 | 9.2 | 0.25 |
| 17 | F | 30 | Active (first episode) | Active | 4 | 17.6 | 0.63 |
| 18 | F | 41 | Active (first episode) | Active | 11 | 16.0 | 0.09 |
| 19 | F | 26 | Active (first episode) | Active | 6 | 5.8 | 1.09 |
| 20 | F | 32 | Inactive | Inactive | 0 | 138.8 | 0.03 |
| 21 | F | 51 | Inactive | Inactive | 0 | 172.2 | 0.06 |
| 22 | F | 54 | Inactive | Inactive | 1 | 329.5 | 0.07 |
| 23 | F | 61 | Inactive | Inactive | 0 | 292.8 | 0.14 |
| 24 | F | 64 | Inactive | Inactive | 0 | 341.0 | 0.54 |
| 25 | F | 60 | Inactive | Inactive | 3 | 427.5 | 0.08 |
| 26 | F | 27 | Inactive | Inactive | 11 | 85.4 | 0.41 |
| 27 | F | 39 | Inactive | Inactive | 4 | 232.4 | 0.18 |
| 28 | F | 62 | Inactive | Inactive | 0 | 115.3 | 0.03 |
| 29 | F | 31 | Inactive | Inactive | 8 | 208.3 | 0.03 |
| 30 | F | 49 | Inactive | Inactive | 0 | 111.6 | 0.22 |
| 31 | F | 68 | Inactive | Inactive | 3 | 485.5 | 0.03 |
| 32 | F | 75 | Inactive | Inactive | 7 | No data | 0.17 |
| 33 | F | 48 | Inactive | Inactive | 4 | 451.2 | 0.53 |
| 34 | F | 45 | Inactive | Inactive | 0 | 126.7 | 0.03 |
| 35 | M | 19 | Inactive | Inactive | 0 | 32.4 | 0.04 |
| 36 | F | 52 | Inactive | Inactive | 0 | 437.7 | 0.03 |
| 37 | F | 38 | Inactive | Inactive | 0 | 223.3 | 0.05 |
| 38 | F | 49 | Inactive | Inactive | 0 | 113.1 | 0.05 |
| 39 | F | 66 | Inactive | Inactive | 0 | 114.0 | 0.09 |
| 40 | F | 63 | Inactive | Inactive | 0 | 433.6 | 0.07 |
| 41 | F | 21 | Inactive | Inactive | 0 | 23.9 | 0.11 |
| 42 | F | 52 | Inactive | Inactive | 4 | 361.5 | 0.03 |
| 43 | F | 34 | Inactive | Inactive | 0 | 88.9 | 0.03 |
| 44 | F | 41 | Inactive | Inactive | 9 | 46.1 | 0.03 |
| 45 | F | 52 | Inactive | Inactive | 3 | 93.1 | 0.25 |
| 46 | F | 48 | Inactive | Inactive | 4 | 126.0 | 0.03 |
| 47 | F | 55 | Inactive | Inactive | 0 | 407.4 | 0.05 |
| 48 | F | 42 | Inactive | Inactive | 7 | 77.3 | 0.03 |
| 49 | F | 40 | Inactive | Inactive | 6 | 53.8 | 0.56 |

NIH, National Institutes of Health; ITAS, Indian Takayasu’s Arteritis Activity Score; CRP, C-reactive protein.
