# Supplementary material for: Vessel Wall Inflammation of Takayasu Arteritis Detected by Contrast-Enhanced Magnetic Resonance Imaging: Association with Disease Distribution and Activity
Source: PLoS One. 2015 Dec 31;10(12):e0145855. doi: 10.1371/journal.pone.0145855 (PMC4700986; doi:10.1371/journal.pone.0145855)
Supplement: S2 Table — LGE, late gadolinium enhancement; SNR, signal-to-noise ratios; CNR, contrast-to-noise ratios. (DOCX) [file pone.0145855.s002.docx]

**S2 Table. Detailed results of qualitative and quantitative analysis for 49 patients.**

| Patient No. | LGE distribution classification | Angiographic classification | LGE distribution larger than Angiographic distribution | preSNR | preCNR | postSNR | postCNR | SNR increment | CNR increment |
| --- | --- | --- | --- | --- | --- | --- | --- | --- | --- |
| 1 | 5 | 5 |  | 13.33 | 11.13 | 50.83 | 42.05 | 37.49 | 30.92 |
| 2 | 5 | 5 |  | 17.06 | 15.43 | 59.43 | 43.05 | 42.37 | 27.63 |
| 3 | 5 | 1 | Yes | 25.23 | 12.99 | 39.41 | 32.66 | 14.18 | 19.67 |
| 4 | 2b | 1 |  | 17.35 | 13.65 | 51.35 | 43.53 | 34.00 | 29.89 |
| 5 | 5 | 2a | Yes | 53.92 | 50.15 | 114.10 | 82.55 | 60.18 | 32.40 |
| 6 | 5 | 1 | Yes | 13.61 | 9.00 | 41.33 | 32.75 | 27.72 | 23.75 |
| 7 | 5 | 1 | Yes | 3.80 | -2.93 | 49.01 | 36.31 | 45.21 | 39.25 |
| 8 | 5 | 2a | Yes | 14.55 | 11.22 | 46.19 | 29.18 | 31.64 | 17.96 |
| 9 | 5 | 5 | Yes | 22.71 | 14.77 | 27.95 | 18.74 | 5.23 | 3.97 |
| 10 | 2b | 2b |  | 35.21 | 28.98 | 91.25 | 49.58 | 56.04 | 20.60 |
| 11 | 5 | 1 | Yes | 20.88 | 18.76 | 42.01 | 33.65 | 21.14 | 14.89 |
| 12 | 5 | 1 | Yes | 12.70 | 5.28 | 53.02 | 29.19 | 40.32 | 23.91 |
| 13 | 5 | 2a | Yes | 8.04 | 3.76 | 117.62 | 88.43 | 109.57 | 84.67 |
| 14 | 5 | 1 | Yes | 10.03 | 1.78 | 28.17 | 25.95 | 18.14 | 24.17 |
| 15 | 2b | 1 | Yes | 11.34 | 8.73 | 35.41 | 24.48 | 24.07 | 15.75 |
| 16 | 2b | 2a | Yes | 17.59 | 13.67 | 43.56 | 30.13 | 25.97 | 16.45 |
| 17 | 5 | 2a | Yes | 7.47 | 3.93 | 48.69 | 43.36 | 41.21 | 39.43 |
| 18 | 5 | 1 | Yes | 31.50 | 25.68 | 31.77 | 13.49 | 0.27 | -12.19 |
| 19 | 5 | 1 | Yes | 9.69 | 5.81 | 49.58 | 35.04 | 39.90 | 29.23 |
| 20 | 5 | 5 |  | 13.08 | 10.74 | 71.61 | 52.90 | 58.53 | 42.16 |
| 21 | 2b | 1 | Yes | 10.47 | 8.12 | 46.60 | 16.72 | 36.14 | 8.61 |
| 22 | 5 | 5 |  | 24.22 | 21.33 | 68.65 | 50.43 | 44.42 | 29.10 |
| 23 | 5 | 1 | Yes | 12.28 | 9.83 | 25.31 | 17.85 | 13.03 | 8.03 |
| 24 | 5 | 2a | Yes | 12.90 | 10.43 | 40.76 | 32.07 | 27.86 | 21.64 |
| 25 | 5 | 1 | Yes | 29.06 | 26.52 | 53.57 | 36.21 | 24.51 | 9.69 |
| 26 | 5 | 3 | Yes | 29.82 | 23.13 | 50.86 | 34.90 | 21.05 | 11.77 |
| 27 | 5 | 2b | Yes | 34.23 | 32.39 | 45.27 | 35.50 | 11.05 | 3.11 |
| 28 | 5 | 2b | Yes | 22.90 | 19.28 | 37.81 | 19.97 | 14.91 | 0.69 |
| 29 | 5 | 2a | Yes | 22.06 | 19.89 | 121.38 | 99.40 | 99.32 | 79.52 |
| 30 | 2b | 2b |  | 15.80 | 12.81 | 61.97 | 41.81 | 46.17 | 29.00 |
| 31 | 5 | 2b | Yes | 3.71 | -2.21 | 23.72 | 17.96 | 20.01 | 20.17 |
| 32 | 5 | 5 |  | 20.85 | -2.38 | 113.71 | 72.67 | 92.86 | 75.04 |
| 33 | 5 | 5 |  | 25.89 | 17.21 | 44.45 | 33.17 | 18.55 | 15.95 |
| 34 | 2a | 5 |  | 14.26 | 11.73 | 36.33 | 10.04 | 22.07 | -1.68 |
| 35 | 5 | 1 | Yes | 17.60 | 11.81 | 12.26 | 9.04 | -5.34 | -2.77 |
| 36 | 2a | 1 | Yes | 15.30 | 13.04 | 36.00 | 15.83 | 20.70 | 2.79 |
| 37 | 5 | 1 | Yes | 19.80 | 16.25 | 42.99 | 29.37 | 23.19 | 13.12 |
| 38 | 5 | 5 |  | 29.35 | 23.53 | 46.84 | 39.30 | 17.49 | 15.78 |
| 39 | 5 | 5 |  | 4.84 | 2.54 | 34.70 | 12.63 | 29.86 | 10.09 |
| 40 | 5 | 1 | Yes | 23.78 | 18.38 | 41.53 | 29.58 | 17.75 | 11.19 |
| 41 | 5 | 1 | Yes | 13.99 | 11.92 | 98.96 | 83.35 | 84.98 | 71.43 |
| 42 | 5 | 5 |  | 22.72 | 15.71 | 44.11 | 27.44 | 21.39 | 11.73 |
| 43 | 5 | 1 | Yes | 11.17 | 8.42 | 40.95 | 27.70 | 29.77 | 19.28 |
| 44 | 5 | 2a | Yes | 21.07 | 2.26 | 45.77 | 32.50 | 24.70 | 30.25 |
| 45 | 5 | 1 | Yes | 14.08 | 9.18 | 73.03 | 49.33 | 58.95 | 40.15 |
| 46 | 5 | 4 | Yes | 16.44 | 8.68 | 74.79 | 57.20 | 58.34 | 48.52 |
| 47 | 5 | 5 |  | 10.65 | 7.20 | 54.62 | 40.85 | 43.97 | 33.65 |
| 48 | 2b | 5 |  | 31.91 | 28.49 | 38.66 | 21.88 | 6.76 | -6.62 |
| 49 | 5 | 2a | Yes | 22.18 | 18.62 | 69.75 | 39.81 | 47.57 | 21.19 |

LGE, late gadolinium enhancement; SNR, signal-to-noise ratios; CNR, contrast-to-noise ratios.
